# Glycemia-based predictors of T2D development in adults with obesity and prediabetes: SURMOUNT-1 post hoc analysis

**DOI:** 10.1210/jendso/bvag190

**Published:** 2026-08-19

**Authors:** Rodolfo J Galindo, Georgios K Dimitriadis, Adam Stefanski, Kieren J Mather, Dachuang Cao, Rhea Lewis, Clare J Lee

**Affiliations:** Division of Endocrinology, Diabetes and Metabolism, University of Miami Miller School of Medicine, Miami, FL 33136, USA; Lilly Corporate Center, Eli Lilly and Company, Indianapolis, IN 46285, USA; Lilly Corporate Center, Eli Lilly and Company, Indianapolis, IN 46285, USA; Lilly Corporate Center, Eli Lilly and Company, Indianapolis, IN 46285, USA; Lilly Corporate Center, Eli Lilly and Company, Indianapolis, IN 46285, USA; Lilly Corporate Center, Eli Lilly and Company, Indianapolis, IN 46285, USA; Lilly Corporate Center, Eli Lilly and Company, Indianapolis, IN 46285, USA

**Keywords:** obesity, prediabetes, GIP/GLP-1, type 2 diabetes, hemoglobin A1c (HbA1c), OGTT (oral glucose tolerance test)

## Abstract

**Objective:**

This post hoc analysis aimed to assess the predictive value of glycemia-based criteria for development of type 2 diabetes (T2D) among adults with obesity and prediabetes in the 3-year SURMOUNT-1 study.

**Methods:**

We evaluated the sensitivity, specificity, and positive and negative predictive value of each of the following exploratory criteria for their ability to correctly identify incident T2D cases in the placebo group: oral glucose tolerance test (OGTT) 1-h glucose ≥155 mg/dL (≥8.6 mmol/L), fasting serum glucose (FSG) ≥ 110 mg/dL (≥6.1 mmol/L), and hemoglobin A1c (HbA1c) ≥ 6% (≥42.1 mmol/mol).

**Results:**

The OGTT 1-h glucose, FSG, and HbA1c based criteria, respectively, had a sensitivity of 0.83, 0.50, and 0.47; specificity of 0.25, 0.87, and 0.69; positive predictive value of 0.15, 0.37, and 0.19; and negative predictive value of 0.91, 0.92, and 0.90. Tirzepatide treatment was associated with a 90% to 99% reduction in new-onset T2D in the high-risk groups, vs 68% to 91% in the standard risk groups using the 3 different criteria.

**Conclusion:**

In this post hoc analysis, we found that OGTT 1-h glucose ≥155 mg/dL (≥8.6 mmol/L) may be a useful criterion to better identify those at high risk of incident T2D. Tirzepatide treatment was highly effective in T2D prevention compared with placebo, regardless of the high-risk criteria used. These findings may guide in the identification of people in whom intensive therapies could be considered to prevent new-onset T2D.

With increasing prevalence of obesity, prediabetes, and undiagnosed type 2 diabetes (T2D), there is an urgency to better identify those at the highest risk of developing T2D and related complications. Glycated hemoglobin (HbA1c) values of 6% to 6.4% (42.1-46.4 mmol/mol) and fasting glucose 110 to 125 mg/dL (6.1-6.9 mmol/L) are associated with higher risk for T2D and diabetic retinopathy and may motivate earlier intervention to prevent T2D (1-3). Oral glucose tolerance test (OGTT) 1 hour glucose ≥155 mg/dL (≥8.6 mmol/L) has been recommended recently by the International Diabetes Federation as a criterion to detect those at high risk of developing T2D, which in turn may allow for early detection, intervention, and improved health outcomes (4-6). However, these glycemic criteria have not yet been examined or implemented widely in adults with obesity and prediabetes.

In this post hoc analysis of the 3-year SURMOUNT-1 trial, we aimed to compare the predictive value of glycemia-based criteria for developing T2D among adults with obesity and prediabetes. We aimed to evaluate the predictive value of glycemia-based criteria for high risk for incident T2D among participants in the placebo group and the differences in T2D prevention between tirzepatide and placebo based on these criteria.

## Research design and methods

### Trial design

SURMOUNT-1 was a 3-year, double-blind, randomized, placebo-controlled clinical trial in participants (N = 1032) with obesity or overweight with at least one weight-related complication and prediabetes. Prediabetes was diagnosed using HbA1c (5.7%-6.4%; 38.8-46.4 mmol/mol), fasting glucose (100-125 mg/dL; 5.6-6.9 mmol/L), and 2-hour glucose values from the OGTT (140-199 mg/dL; 7.8-11.0 mmol/L). At least 2 abnormal tests were required to diagnose prediabetes. Participants were randomly assigned in a 1:1:1:1 ratio to once-weekly 5, 10, or 15 mg tirzepatide or matched placebo. The diagnosis of diabetes was based on American Diabetes Association (ADA) guidelines and confirmed by an independent adjudication committee (7, 8).

### Post hoc analyses

Data of the 176-week treatment period from all randomized participants who received at least 1 dose of study drug (N = 1032; n = 270 placebo; n = 762 tirzepatide) were used for analysis, with tirzepatide dose groups pooled. For each high-risk criterion (baseline OGTT 1-h glucose ≥155 mg/dL [≥8.6 mmol/L], fasting serum glucose [FSG] ≥ 110 mg/dL [≥6.1 mmol/L], HbA1c ≥ 6% [≥42.1 mmol/mol]), the associated odds of developing T2D were evaluated using logistic regression with fixed effects of treatment (tirzepatide vs placebo), high-risk (yes vs no), sex, and treatment by high-risk interaction. A multivariate logistic regression analysis was performed to include all 3 high-risk criteria and other potential risk factors (eg, age, body mass index [BMI]). Classification performance of these criteria was evaluated using sensitivity and specificity in the placebo group and not in the tirzepatide-treated group given the small event number (n = 5) in the tirzepatide-treated group. Statistical significance threshold was 0.05, and for interaction 0.10. A Venn diagram was created to illustrate all incident T2D cases identified using the glycemic criteria.

## Results

### Baseline characteristics across all included participants

Demographics and baseline characteristics are summarized in Table 1. Using all 3 criteria combined, those in the high-risk group had significantly greater waist circumference than those in the standard risk group. Using the HbA1c and FSG criteria, those in the high-risk groups were significantly older. Using the OGTT 1-h glucose and FSG criteria those in the high-risk groups had significantly higher fasting insulin and significantly higher triglycerides than those in the standard risk group.

**Table 1:** Demographic and baseline characteristics of high-risk and standard risk participants

	Criterion #1		Criterion #2		Criterion #3	
	OGTT 1-h glucose ≥155 mg/dL (≥8.6 mmol/L) (N = 790)	OGTT 1-h glucose <155 mg/dL (<8.6 mmol/L) (N = 236)	FSG ≥110 mg/dL (≥6.1 mmol/L) (N = 168)	FSG <110 mg/dL (<6.1 mmol/L) (N = 864)	HbA1c ≥6% (≥42.1 mmol/mol) (N = 323)	HbA1c <6% (<42.1 mmol/mol) (N = 709)
Age, y	48.3 (11.8)	47.4 (12.1)	50.9 (11.5)*	47.6 (11.8)	50.7 (11.4)*	47.0 (11.9)
Gender, female, n (%)	488 (61.8)	169 (71.6)	97 (57.7)	562 (65.0)	207 (64.1)	452 (63.8)
Race, n (%)						
White	583 (73.8)	172 (72.9)	145 (86.3)*	613 (70.9)	222 (68.7)*	536 (75.6)
Black	49 (6.2)	27 (11.4)	2 (1.2)*	75 (8.7)	45 (13.9)*	32 (4.5)
Asian	87 (11.0)	18 (7.6)	12 (7.1)*	93 (10.8)	33 (10.2)*	72 (10.2)
Multiple	11 (1.4)	2 (0.8)	0	13 (1.5)	7 (2.2)	6 (0.8)
Body weight, kg	107.9	105.3	111.0 (24.2)*	106.6 (23.1)	108.8 (24.3)	106.7 (22.9)
BMI, kg/m^2^	38.9 (7.1)	38.3 (7.2)	39.8 (7.2)*	38.6 (7.1)	39.4 (7.5)	38.5 (6.9)
BMI category, n (%)						
<30	39 (4.9)	12 (5.1)	5 (3.0)	46 (5.3)	18 (5.6)	33 (4.7)
≥30 to <35	229 (29.0)	75 (31.8)	42 (25.0)	265 (30.7)	82 (25.4)	225 (31.7)
≥35	522 (66.1)	149 (63.1)	121 (72.0)	553 (64.0)	223 (69.0)	451 (63.6)
Waist circumference, cm	117.2 (15.7)*	114.5 (15.3)	119.9 (16.6)*	115.9 (15.3)	118.4 (15.4)*	115.7 (15.6)
HbA1c, %	5.8 (0.3)*	5.7 (0.3)	5.8 (0.4)	5.7 (0.3)	6.1 (0.2)*	5.6 (0.3)
HbA1c, mmol/mol	39.7 (3.7)*	38.9 (3.6)	40.4 (4.5)	39.3 (3.5)	42.7 (2.3)*	38.0 (3.3)
Waist to height ratio						
FSG, mg/dL	101.8 (9.8)*	99.9 (8.7)	109.0 (9.5)*	99.8 (8.8)	102.1 (10.7)	101.0 (9.0)
FSG, mmol/L	5.7 (0.5)*	5.5 (0.5)	6.1 (0.5)*	5.5 (0.5)	5.7 (0.6)	5.6 (0.5)
Fasting insulin (mIU/L)	17.2 (13.2)*	13.9 (9.1)	18.2 (11.1)*	16.1 (12.7)	16.7 (9.5)	16.3 (13.6)
OGTT, mg/dL						
0 hour	104.7 (9.3)*	101.2 (8.1)	111.0 (9.3)*	102.5 (8.4)	104.3 (9.8)	103.7 (8.9)
1 hour	196.9 (27.5)*	131.5 (19.2)	200.1 (42.1)*	178.3 (35.8)	190.1 (37.9)*	178.2 (37.2)
2 hour	139.9 (30.7)*	115.0 (27.7)	136.9 (33.3)	133.7 (31.6)	138.8 (32.6)*	132.2 (31.3)
OGTT, mmol/L						
0 hour	5.8 (0.5)*	5.6 (0.5)	6.2 (0.5)*	5.7 (0.5)	5.8 (0.5)	5.8 (0.5)
1 hour	10.9 (1.5)*	7.3 (1.1)	11.1 (2.3)*	9.9 (2.00)	10.6 (2.1)*	9.9 (2.1)
2 hour	7.8 (1.7)*	6.4 (1.5)	7.6 (1.9)	7.4 (1.8)	7.7 (1.8)*	7.3 (1.7)
SBP, mmHg	125.8 (12.7)	124.8 (12.9)	126.5 (12.8)	125.4 (12.7)	126.0 (12.8)	125.4 (12.7)
Current smoker, n (%)	88 (11.1)	28 (11.9)	18 (10.7)	98 (11.3)	42 (13.0)	74 (10.4)
Lipids, mg/dL						
Triglycerides	157.7 (87.0)*	133.8 (61.5)	164.7 (98.6)*	149.8 (78.7)	148.0 (71.5)	154.2 (86.9)
HDL	47.4 (11.9)*	49.4 (12.8)	45.7 (10.7)	48.3 (12.4)	48.3 (11.8)	47.6 (12.3)
LDL	113.7 (32.8)	114.7 (35.0)	109.7 (30.9)	114.5 (33.8)	112.8 (33.0)	114.2 (33.5)
UACR, g/kg	28.3 (172.5)	24.7 (176.9)	54.6 (300.5)*	22.0 (134.4)	23.3 (91.7)	29.2 (199.4)
Estimated GFR, mL/min/1.73 m^2^	95.0 (17.3)	95.7 (20.1)	91.8 (18.3)*	95.7 (17.8)	94.0 (17.7)	95.6 (18.1)
Treatment assignment, n						
Tirzepatide	585	172	119	643	234	528
Placebo	205	64	49	221	89	181

Data are mean ± SD or *n* (%). * *P* < .05 High-risk vs standard risk based on Chi-square or ANOVA. Participants assigned to tirzepatide were treated with 5 mg, 10 mg, or 15 mg tirzepatide. For these analyses these treatment groups were pooled. OGTT 1-h glucose, N = 1026; FSG, N = 1032; HbA1c, N = 1032.

Abbreviations: BMI, body mass index; FSG, fasting serum glucose; GFR, glomerular filtration rate; HbA1c, glycated hemoglobin; HDL, high-density lipoprotein; LDL, low-density lipoprotein; OGTT, oral glucose tolerance test; SBP, systolic blood pressure; UACR, urine albumin-to-creatinine ratio.

### Developing T2D: tirzepatide vs placebo

**Figure 1:**
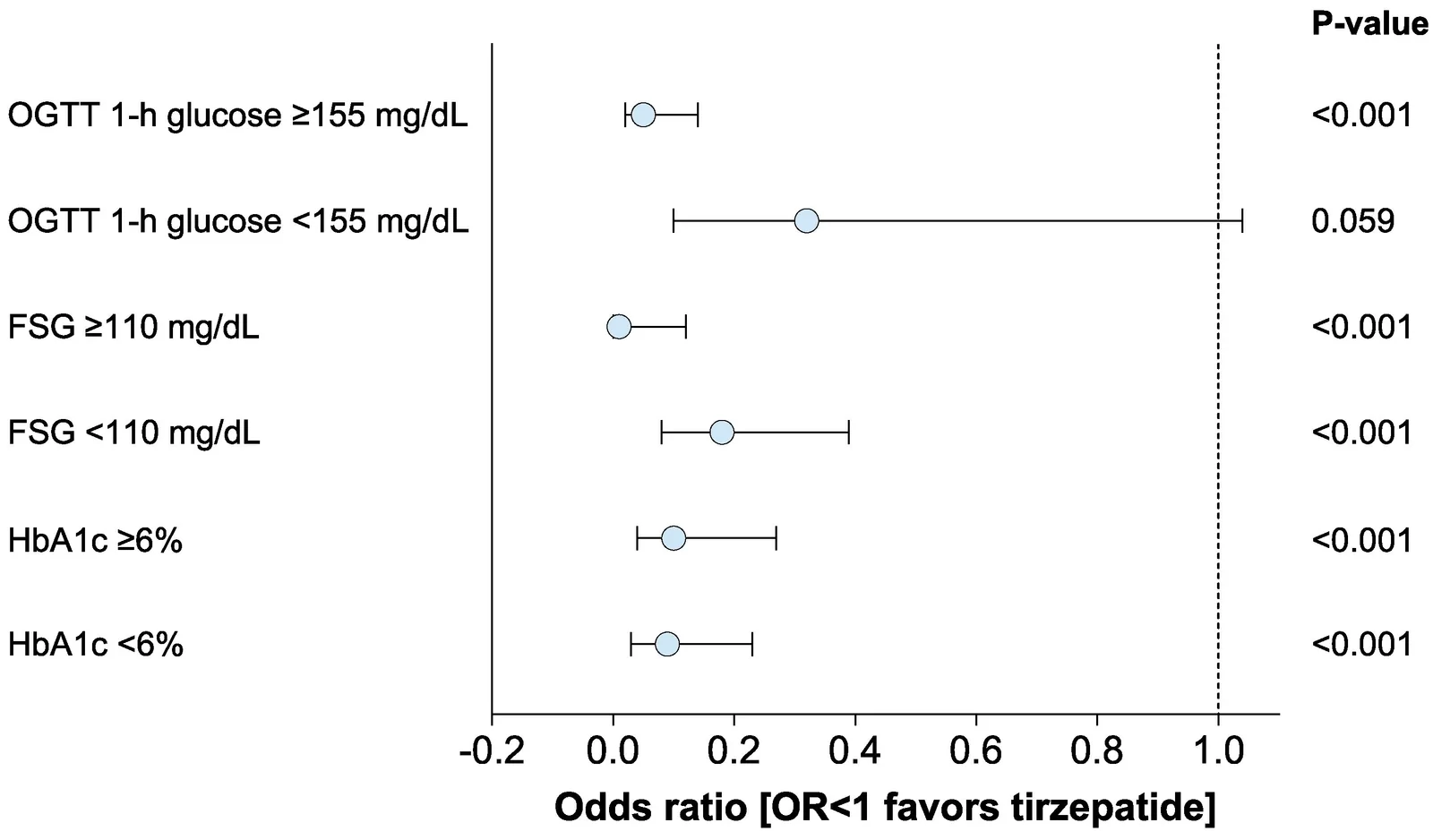
Forest plot for odds ratio of incidence of T2D of those treated with tirzepatide vs placebo within risk groups. OGTT 1-h glucose: 155 mg/dL = 8.6 mmol/L; FSG: 110 mg/dL = 6.1 mmol/L; HbA1c: 6% = 42.1 mmol/mol.

The incidence of T2D was low in those treated with tirzepatide regardless of risk group across all glycemic criteria (high-risk, 0-5 cases; standard risk, 5-10 new cases) (Table 2, Fig. 1). The incidence of T2D was numerically greater in the placebo group (high-risk, 17-30 new cases; standard risk, 6-19 new cases) (Table 2).

**Table 2:** Onset of T2D

Criterion	Risk group	# Incident T2D	Treatment	N	Incident T2D n (%)	Interaction *P* value
#1	OGTT 1-h glucose ≥155 mg/dL (≥8.6 mmol/L)	35	Tirzepatide	585	5 (0.9)	.024
			Placebo	205	30 (14.6)	
	OGTT 1-h glucose <155 mg/dL (<8.6 mmol/L)	11	Tirzepatide	172	5 (2.9)	
			Placebo	64	6 (9.4)	
#2	FSG ≥110 mg/dL (≥6.1 mmol/L)	18	Tirzepatide	119	0	.031
			Placebo	49	18 (36.7)	
	FSG <110 mg/dL (<6.1 mmol/L)	28	Tirzepatide	643	10 (1.6)	
			Placebo	221	18 (8.1)	
#3	HbA1c ≥ 6% (≥42.1 mmol/mol)	22	Tirzepatide	234	5 (2.1)	.849
			Placebo	89	17 (19.1)	
	HbA1c < 6% (<42.1 mmol/mol)	24	Tirzepatide	528	5 (1.0)	
			Placebo	181	19 (10.5)	

Abbreviations: FSG, fasting serum glucose; HbA1c, glycated hemoglobin; OGTT, oral glucose tolerance test; T2D, type 2 diabetes.

Tirzepatide treatment was associated with a significant reduction in incident T2D compared with placebo in 5 out of 6 glycemic risk subgroups shown in Fig. 1. Further, the degree of risk reduction with tirzepatide vs placebo appeared similar regardless of the high vs standard risk glycemic criteria (Fig. 1).

### Developing T2D: high vs standard risk group

Overall, individuals in the high-risk group were at higher risk of progression to T2D. However, this risk varied by study treatment. The magnitude of risk reduction with tirzepatide treatment compared with placebo in incident T2D was significantly greater in the high vs standard risk group for OGTT 1-h glucose (*P* = .024) and for FSG (*P* = .031) but not for HbA1c (*P* = .849) (Table 2).

### Performance of different glycemia-based diagnostic criteria in the placebo group

**Figure 2:**
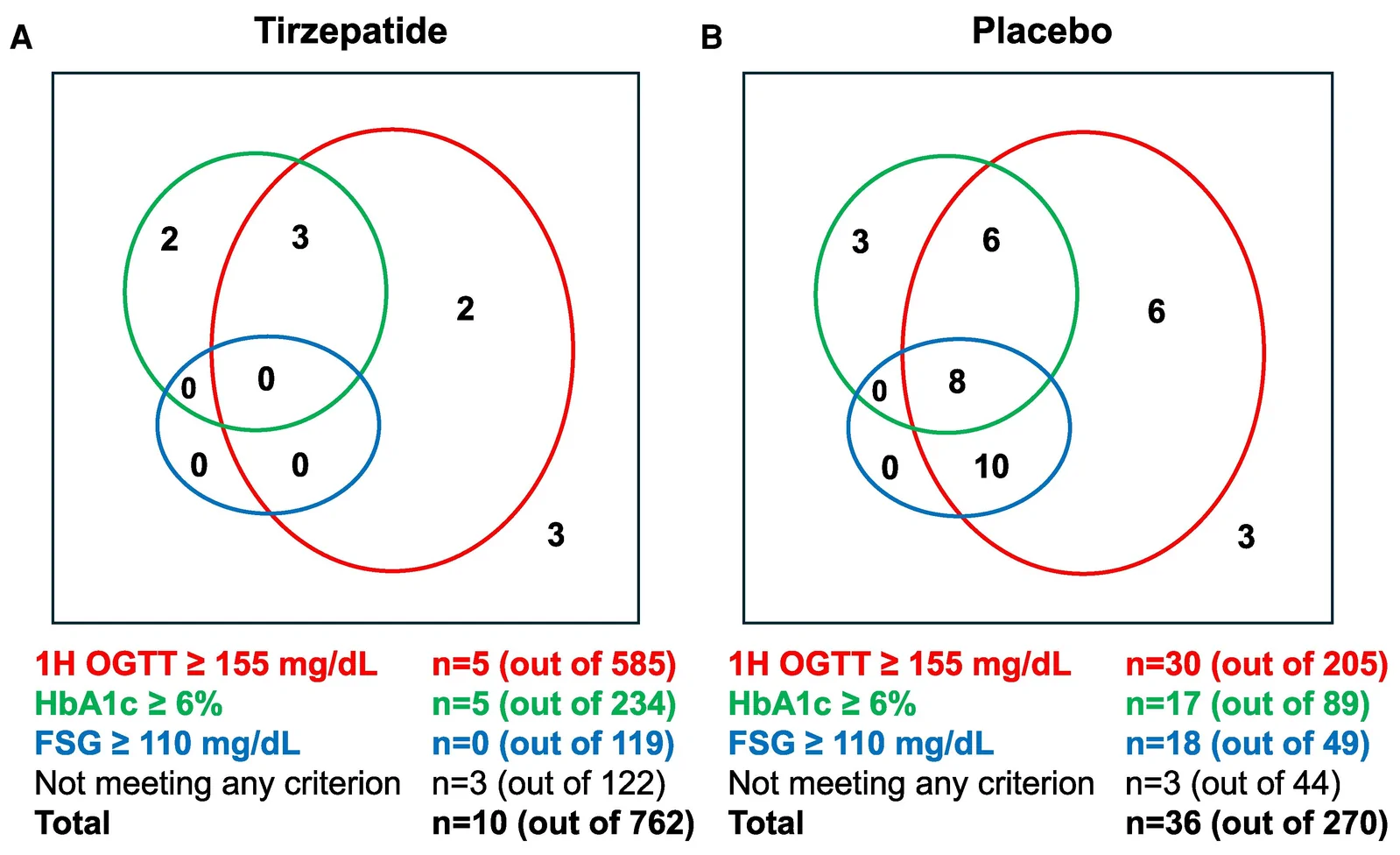
Venn diagram of incident T2D during 176 weeks by the glycemia-based high-risk criteria. For both (A) Tirzepatide and (B) Placebo groups, each circle represents the participants meeting the given high-risk criterion; the area outside the 3 circles represents the participants not meeting any of the high-risk criteria. Number (n) is the number of T2D cases among the represented participants. OGTT 1-h glucose: 155 mg/dL = 8.6 mmol/L; FSG: 110 mg/dL = 6.1 mmol/L; HbA1c: 6% = 42.1 mmol/mol.

In the placebo group, the OGTT 1-h glucose criterion had the highest sensitivity of 0.83, yet the lowest specificity of 0.25 and it correctly identified the greatest proportion of participants being high-risk (Fig. 2). In contrast, the FSG criterion had a low sensitivity of 0.50, and the highest specificity of 0.87. The HbA1c criterion had sensitivity of 0.47 and specificity of 0.69. For OGTT 1-h glucose, FSG, and HbA1c criteria, the positive predictive values were 0.15, 0.37, and 0.19 respectively, and negative predictive values were 0.91, 0.92, and 0.90 respectively.

## Discussion

In this post hoc analysis of people with obesity and prediabetes in the 3-year SURMOUNT-1 trial, we evaluated the utility of several glycemia-based criteria to identify individuals at risk of progression to T2D. The OGTT 1-h glucose cutoff of 155 mg/dL (8.6 mmol/L) had the highest sensitivity (0.83) in correctly identifying 76% (n = 35) of the incident T2D cases albeit with relatively low specificity (0.25). In contrast, the FSG cutoff of 110 mg/dL (≥6.1 mmol/L) and HbA1c cutoff of 6% (42.1 mmol/mol) had lower sensitivity (0.50 and 0.47, respectively) and each identified fewer cases of incident T2D (39%, n = 18% and 48%, n = 22 respectively). All 3 glycemia-based criteria had high negative predictive values and low positive predictive values, likely related to the relatively low number of cases of incident T2D in this study.

In the placebo group, the majority (67%, n = 24) of those who developed T2D in the study met 2 or more of the high-risk criteria explored in this analysis. Further, all cases of incident T2D per FSG cutoff also met the OGTT 1-h glucose cutoff and most (82%) of those who met the HbA1c cutoff also met the OGTT 1-h glucose cutoff. Put another way, HbA1c cutoff alone would have missed more than half (53%, n = 19) of the incident T2D cases and adding the FSG cutoff would have helped detect 10 additional cases out of the 19 missed cases. Adding the OGTT 1-h glucose test would have helped further detect 6 more cases out of the remaining 9 missed cases.

These results support recent recommendations from the International Diabetes Federation Position Statement to use OGTT 1-h glucose ≥ 155 mg/dL (8.6 mmol/L) as a predictive method to detect progression to T2D early, in high-risk individuals (4). As described previously, using the OGTT 1-h glucose cutoff may allow clinicians to avoid misclassification. In turn, this approach could also allow for an early identification of people at risk of progression to T2D and guide prevention strategies including intensive interventions that combine lifestyle intervention with pharmacotherapy (4, 9).

Tirzepatide treatment was associated with a substantial reduction in the risk of progression to T2D compared with placebo regardless of the high-risk criteria used. Tirzepatide treatment over a period of 3 years was associated with 90% to 99% reduction in the risk of developing the T2D in the high-risk groups vs 68% to 91% risk reduction in the standard risk groups using the 3 different criteria. In the high-risk group as defined by the OGTT 1-h glucose or FSG cutoff, the magnitude of risk reduction with tirzepatide vs placebo was significantly greater compared with the standard risk group. As expected, the characteristics of the high-risk group were notable for features consistent with elevated cardiometabolic risk, including higher waist circumference, older age, higher fasting insulin, and triglycerides. In the setting of limited resources and rising epidemic of obesity and prediabetes, these findings highlight the cohort with the highest potential risk of progression from prediabetes to T2D, in which intensive interventions, such as tirzepatide treatment, could be considered (10).

Strengths of this post hoc analysis include thorough assessment of glycemic status at baseline and at the end of the trial, which included 2-hour OGTT in all participants, long-term data (over 3 years) across individuals with obesity and prediabetes, and adjudication-confirmed new-onset T2D. Limitations include the relatively low number of new-onset T2D events, potential burden and availability of OGTT that may limit generalizability, and the exploratory nature of the limited number of glycemia-based criteria examined in this post hoc analysis. We have focused on the most commonly used glycemic metrics while recognizing that there are other indices currently under investigation for T2D prediction (11, 12).

In summary, we evaluated several criteria that identify risk for incident T2D in this post hoc analysis of adults with obesity and prediabetes in the 3-year SURMOUNT-1 trial. We found that the OGTT 1-h glucose cutoff of 155 mg/dL (8.6 mmol/L) had the highest sensitivity compared with the HbA1c and FSG criteria, while all 3 criteria had relatively low specificity. Tirzepatide treatment was associated with greater prevention of new-onset T2D among adults living with obesity and prediabetes compared with placebo, and the degree of risk reduction with tirzepatide vs placebo was similar across these criteria. Combined, these results support further studies of OGTT 1-h glucose as a potential criterion to better identify those at high risk of developing T2D among individuals with obesity and prediabetes, which in turn may guide prevention strategies including intensive interventions.

## Data Availability

Some or all datasets generated during and/or analyzed during the current study are not publicly available but are available from the corresponding author on reasonable request. Eli Lilly and Company provides access to all individual participant data collected during the trial, after anonymization, with the exception of pharmacokinetic or genetic data. Data are available to request 6 months after the indication studied has been approved in the US and EU and after primary publication acceptance, whichever is later. No expiration date of data requests is currently set once data are made available. Access is provided after a proposal has been approved by an independent review committee identified for this purpose and after receipt of a signed data sharing agreement. Data and documents, including the study protocol, statistical analysis plan, and blank or annotated case report forms, will be provided in a secure data sharing environment. For details on submitting a request, see the instructions provided at www.vivli.org.

## Abbreviations

BMI: body mass index
FSG: fasting serum glucose
HbA1c: glycated hemoglobin
OGTT: oral glucose tolerance test
T2D: type 2 diabetes
